# Severe acute malnutrition’s recovery rate still below the minimum standard: predictors of time to recovery among 6- to 59-month-old children in the healthcare setting of Southwest Ethiopia

**DOI:** 10.1186/s41043-022-00331-9

**Published:** 2022-11-04

**Authors:** Seyum Ebissa Eyi, Gebiso Roba Debele, Efrem Negash, Kebebe Bidira, Debela Tarecha, Kabtamu Nigussie, Mohammedamin Hajure, Mohammedjud Hassen Ahmed, Bilisumamulifna Tefera Kefeni

**Affiliations:** 1 Department of Public Health, College of Health Sciences, Mattu University, Mattu, Ethiopia; 2 Department of Psychiatry, College of Health Sciences, Mattu University, Mattu, Ethiopia; 3 Department of Health Informatics, College of Health Sciences, Mattu University, Mattu, Ethiopia; 4 Department of Nursing, College of Health Sciences, Mattu University, Mattu, Ethiopia; 5 Department of Psychiatry, College of Health and Medical Sciences, Haramaya University, Harar, Ethiopia

**Keywords:** Time to recovery, Child 6–59 months, Severe acute malnutrition, Southwest Ethiopia

## Abstract

**Background:**

Despite currently available, scientifically proven treatments and national guideline, the SAM recovery rate is still considerably behind expectations, and it continues to have a devastating impact on under-five children. Identifying predictors of time to recovery might help to reach the minimal criterion established by the WHO and the national Sphere which decreases child mortality. Therefore, the current study assessed time to recovery and its predictors among children aged 6–59 months admitted with SAM in the Healthcare Setting of Southwest Ethiopia, 2021.

**Methods:**

An institutional-based multicenter retrospective follow-up study was conducted on 486 children aged 6 to 59 months admitted with SAM cases. Data were entered into Epi-Data version 4.6 and exported to Stata version 14 for further analysis. Cox–Snell residual plot was used to assess the final model’s overall goodness of fit. Finally, a significant predictor of time to recovery was identified using Weibull survival regression model, at 0.05 significance level.

**Result:**

Overall, 68.72 (95% CI 64.8, 73) of the children recovered and 4.32% died. The overall incidence density was 3.35/100-person day. Independent predictors of time to recovery were, starting complementary feeding at six months (AHR = 1.44; 95%, CI 1.073, 1.935), pneumonia at baseline (AHR = 1.33, 95%, CI 1.049, 1.696), amoxicillin (AHR = 1.31, 95%, CI 1.021, 1.685), and folic acid supplementation (AHR = 1.82, 95% CI 1,237, 2.665).

**Conclusion:**

The recovery from SAM at study area after a maximum of 60 days of treatment was below the accepted minimum standard. Complementary feeding, pneumonia, treated by amoxicillin, and folic acid supplementation were predictors of time to recovery. Therefore, providing folic acid and amoxicillin for those in need as well as the earliest possible treatment of concomitant conditions like pneumonia is highly recommended.

**Supplementary Information:**

The online version contains supplementary material available at 10.1186/s41043-022-00331-9.

## Introduction

Malnutrition refers to energy and/or nutrient deficits, excesses, or imbalances in a person’s diet [1]. Depending on the level of wasting and the presence of edema, acute malnutrition is categorized into severe and moderate [2]. The presence of nutritional edema, very low weight for height, or evident severe wasting are all signs of severe acute malnutrition (SAM). When the mid-upper arm circumference (MUAC) is less than 11 cm, it is also an indicative of SAM [3, 4].

Despite the fact that stunting has been on the decline since 2000, wasting continues at alarming rates [5]. An estimated 555 million children under the age of five live in the world, with 52 million of them suffering from acute malnutrition and over 19 million being severely wasted [6]. Over 90% of people suffering from acute malnutrition live in developing countries, particularly in Sub-Saharan Africa (SSA) and Southeast Asia [7]. Despite a slight drop from 12 to 10% over the last 15 years, Ethiopia continues to have one of the highest rates of SAM [8]. The prevalence of wasting was 9.9% of which 2.9% are severely wasted [9]. More than 50% of all child deaths globally are either directly or indirectly related to malnutrition [10]. It also increases the incidence and severity of common diseases in children and slows their recovery [11]. A child with SAM has a nine times higher risk of dying than a child who is well-nourished [12]. Acute malnutrition, or wasting, exclusively is an attributable cause of 12.6% of the 6.9 million deaths among children under 5 years old [13].

In Ethiopia, over 25,000 children with SAM are admitted to hospitals each month [14] and it accounts for 20% of pediatric hospital admissions [15]. According to recent studies, recovery rates for SAM patients receiving inpatient care according to the world health organization (WHO) protocol ranged from 33.6 to 88.4% [16–19]. Recent studies conducted in Ethiopia revealed that the recovery rates fall short of the minimal (> 75%) criterion established by the WHO and the national Sphere for judging the performance of therapeutic feeding programs [8, 20–22].

Standardized guidelines for the care of SAM patients have been developed by the WHO [23] and Ethiopia [24, 25] as part of the endeavor to raise the level of inpatient care for severely malnourished children and lower case fatality rates. Additionally, since 2009, Ethiopia has adopted and put into practice national and international commitments including the Seqota Declaration to end all forms of malnutrition [17]. By following these guidelines, the case fatality rate has significantly decreased [21]. However, the SAM recovery rate is still considerably behind expectations, and it continues to have a devastating impact on children under the age of five [26].

Different previous studies identified anemia, tuberculosis (TB), deworming supplementation, malaria, pneumonia, history of bottle feeding, and vaccination status as the most common predictors of time to recovery (increase recovery time) from SAM [8, 20–22, 27, 28]. As of today, evidences show that recovery rate from SAM among children admitted to therapeutic feeding center (TFC) is still lower than the recommended WHO and national Sphere standard. So, to prevent complications and enhance recovery rate due emphasis should be given in improving early detection and treatment of severely malnourished children in Ethiopia. However, the existing evidence on predictors of time to recovery from SAM in children 6–59 months is not enough to halt this problem. In addition, the majority of studies had smaller sample size and unable to clearly show the effects of predictors on time to recovery. Identifying those predictors using adequate sample might help to reach the minimal (> 75%) criterion established by the WHO and the national Sphere.

Thus, this study was aimed to determine the time to recovery from SAM and its predictors among children 6–59 months of age admitted at hospitals of Buno Bedele and Ilu Aba Bor zones, southern Ethiopia.

## Methods and materials

### Study area, design and study period

An institutional-based multicenter retrospective follow-up study was conducted on SAM cases enrolled at the hospitals from September 09, 2017, to April 04, 2021, for 3 years and half. The study was conducted at 3 hospitals: Darimu Primary Hospital (DPH), Bedele General Hospital (BGH), and Mettu Karl Comprehensive Specialized Hospital (MKCSH), Oromia Regional State, South West Ethiopia. DPH currently serves a total of 434,158 people from two districts, with 2269 pregnant women and 10,040 under-five children scheduled for services each year, while MKCSH serves a total of 1.2 million people, with 6270 pregnant women and 27,750 under-five children scheduled for services. ways, BGH serves a total of 841,158 patients annually, including 4395 pregnant women and 19,452 children under the age of five.

### Population and exclusion criteria

All children aged 6–59 months who were treated for SAM at the inpatient therapeutic feeding center of DPH, BGH, and MKCSH was the source population, whereas all children aged 6–59 months admitted with SAM at the inpatient TFC of the hospitals from September 09, 2017, to April 04, 2021, were our study population. All SAM cases with congenital problems, transferred out cases, other causes of edema, and readmitted cases within the study period were excluded.

### Sample size and sampling procedure

#### Size determination

The sample size was calculated using the Schoenfeld formula using Stata software version 14 considering predictors significantly associated with the time to recovery from SAM from prior studies.

$E=\frac{{\left(\frac{Z\alpha }{2}+Z\beta \right)}^{2 }}{P1P2{\left(lnHR\right)}^{2}}$ and $n=\frac{E}{P\left(E\right)}$ = Schoenfeld formula for manual calculation.

where p1 is the proportion of subjects in the exposure group, E is the number of required events, n is the sample size, and HR is the hazard ratio of selected covariates. We computed the sample size by taking the hazard ratio for covariates significantly associated with time to recovery from SAM from studies in the northwest [8] and southern [20] Ethiopia. The sample size computation is summarized in Table 1. Then, 10% of withdrawal probability was considered making final sample size of 499.

**Table 1:** Minimum sample size for covariates associated with time to recovery from SAM, 2021

Assumption	Variables	Hazard ratio (HR)	Probability of event	N(Event)
Type I error = 0.05	Anemia	1.66	0.658	207 (123)
Power = 80%	Malaria	1.54	0.658	285 (169)
Withdrawal probability = 0.1	F100 intake	0.728	0.694	499 (312)

#### Sampling technique

**Fig. 1:**
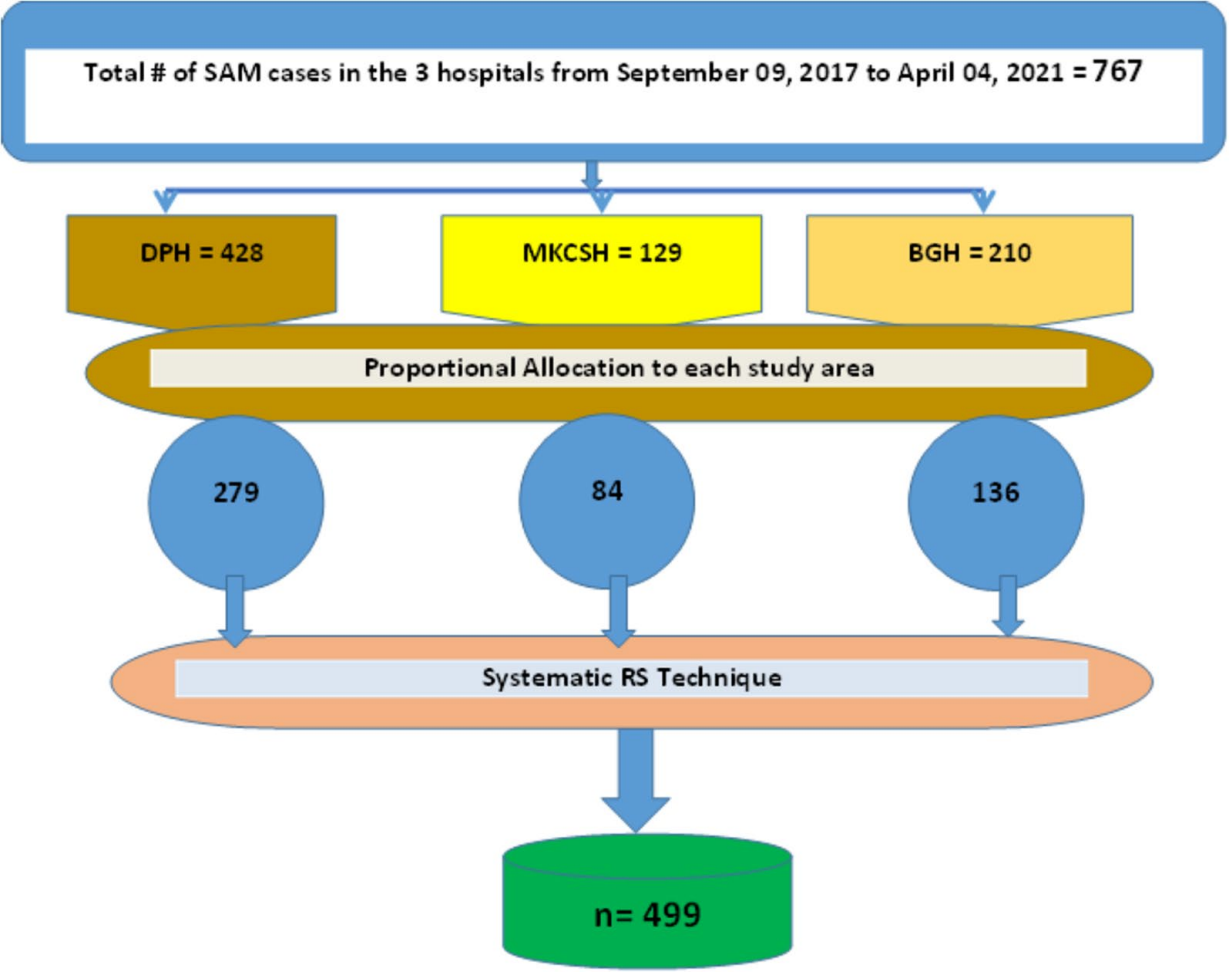
Schematic presentation of sampling procedure for predictors of time to recovery from SAM among age 6–59 months children admitted at TFC of Southwest Ethiopia

Eligible cases were selected using the existing medical registration numbers using a systematic random sampling technique to generate the required sample size, starting from the most recent month and going backward, based on the sequence of medical card numbers. The total sample size for each hospital was allocated proportionally, and the schematic is presented in Fig. 1

### Study variables

#### Dependent variable

Time to recovery from SAM

#### Independent variables

*Socio-demographic and admission characteristics* Referral address (place of treatment), stabilization center, fully vaccination to age, mixed feeding, NG tube feeding, breast feeding status, bottle feeding, received play stimulation, child age, sex, admission and discharge date residence, admission season.

*Therapeutic foods administered* Plumpy nut, F75 intake, F100 intake, not entering phase 2 on day 10 and being admitted to referral hospitals.

*Routine medication and treatment* Vitamin A supplement, use of oral antibiotics (amoxicillin), deworming, special medications (IV fluid, ResoMal, blood transfusion, IV antibiotics).

Anthropometric: Daily weight gain, MUAC, not lose edema within four days of inpatient treatment.

*Medical Comorbidities* TB, Human immune deficiency virus (HIV), pneumonia, anemia, malaria, diarrhea, septic shock, altered body temperature, nutritional status (marasmus, stunting, kwashiorkor dermatosis, marasmus–kwashiorkor).

### Operational definitions of variables

- *Recovered* children whose medical records were classified as cured or recovered after overcoming medical complications and edema and achieving and maintaining MUAC (12.5 cm) and WFH (85 percent) of the median WHO growth chart reference [29, 30].
- *Time to recovery* is the period of time (in days) between the child’s admittance and SAM recovery [31].
- *Censored* includes those who were referred (for actions taken against medical advice), non-responders, defaulters, and those whose demise is noted [28]**.**
- *Non-responder* is a patient who has not met the requirements for discharge after 60 days of inpatient care [20].
- *Defaulters* those who cease therapy before the child is cured or lost with no known condition, or when the patient does so before making a full recovery [8]**.**

### Data collection and quality assurance

Structured data extraction tools were adapted from the Federal Ministry of Health of Ethiopia Protocol for the Management of SAM 2013 [24], and reviewing relevant literature [20–22, 28, 32, 33]. Data were extracted for one month by six [6] BSc nurses after receiving intensive training.

The technique for data collection is daily extraction from the patient medical record and SAM treatment registry retrospectively. The supervisors strictly followed the overall activities of the data collection on a daily basis to ensure the completeness of the questionnaire and to give further clarification and support to data collectors.

A two-day training on the purpose of the study, pertinent ethical issues, the protocols to be followed, how to extract data from medical records, what to extract, and how to internalize the context of each question in the checklist was given to the selected data collectors and supervisors. The tool was preliminary reviewed using 5% of the samples outside the study area. Once the checklist’s adequacy had been determined, variables for which there were no data were left off of it. The appointed supervisors performed routine checks on all tools to ensure data completeness, clarity, and consistency.

### Data processing and statistical analysis

Epi-Data version 4.6 was used for the data entry, which was subsequently exported to Stata version 14 for data cleaning and analysis. The study population was described using descriptive statistics like graphical methods, means, medians, interquartile ranges, percentages, frequencies, and standard deviations. We used the mean and standard deviation (SD) for normally distributed continuous data. The total initial population was used as the denominator and the number of newly admitted SAM children as the numerator to determine the cumulative incidence of recovery from SAM. The ratio of the number of new cases to the number of patient-months at risk was used to compute incidence density.

The Kaplan–Meier (KM) method was used to estimate the time to SAM recovery, and the Log-rank test was performed to compare survival times among categories of categorical factors. Proportional hazard assumption (PHA), which are believed to be satisfied at a p value greater than 0.05, was examined using the Schoenfeld residuals approach (using both a global test and a test for each variable). The variance inflation factor (VIF) was used to test for multicollinearity. The variables with p value less than 0.25 in bivariate Weibull regression analysis were chosen as potential candidates for the multivariate Weibull regression model. Based on the Akaike information criteria (AIC), the best-fitting survival model was chosen. The Cox–Snell residual plot was used to assess the final model’s overall goodness of fit. Finally, multivariable Weibull regression model was used to identify significant predictors of time to recovery from SAM at 0.05 significance level.

## Results

### Socio-demographic and admission characteristics

From a total of 499 SAM records, 486 (97.6%) were used for the final analysis after excluding 13 records with incomplete data. More than half (53.7%) of the children were male, and 33.3% were between the ages of 6 and 11 months. The mean age of the study participants was 19 months, with a standard deviation of 13.99 months. The majority of them (84.4%) were from rural areas. Almost half (51.6%) of the children had a history of breastfeeding at their age, while 43.4%, 27.8%, and 47.5% had a history of supplementary, NG tube, and bottle feeding, respectively. Over half (53.5%) of the children tested received play and stimulation, whereas 24.1% received play and vaccinations (Table 2).

**Table 2:** Socio-demographic and characteristics of SAM children admitted to TFC of DPH, BGH, and MKCSH, from September 09, 2017, to April 04, 2021

Characteristic	Category	Number	Percent
Age of the child	6–11 months	162	33.3
	12–23 months	155	31.9
	24–35 months	86	17.7
	36–47 months	37	7.6
	48–59 months	46	9.5
Sex of the child	Female	225	46.3
	Male	261	53.7
Residence	Rural	410	84.4
	Urban	76	15.6
Exclusive breast feeding	Yes	251	51.6
	No	235	48.4
Complementary feeding	Yes	211	43.4
	No	275	56.6
NG tube feeding	Yes	135	27.8
	No	351	72.2
Bottle feeding	Yes	231	47.5
	No	255	52.5
Child got Play and stimulation	Yes	117	24.1
	No	369	75.9
Fully immunized	Yes	260	53.5
	No	226	46.5

### Medical complications and clinical features of SAM

According to Table 3, fever was the most prevalent sign of an infection at the time of admission (46.7%), whereas the most frequent medical comorbidities among SAM children at the time of admission were cough (45.9%), diarrhea (42.6%), pneumonia (38.3%), vomiting (32.7%), and dehydration (25.7%). The other comorbidities discovered were anemia (11.3%), malaria (11.1%), blood in the stool (4.3%), tuberculosis (3.7%), measles (3.3%), septic or hypovolemic shock (2.1%), and HIV infection (1.6%). Regarding edema, 30.1% of the children admitted to the treatment facility had it at the time of their admission.

**Table 3:** Medical complications and clinical features of SAM children admitted to TFC of DPH, BGH, and MKCSH, from September 09, 2017, to April 04, 2021

Variable	Category	Frequency	%
Dehydrated	Yes	125	25.7
	No	361	74.3
Blood in the stool	Yes	21	4.3
	No	465	95.7
Vomiting	Yes	159	32.7
	No	327	67.3
Cough	Yes	223	45.9
	No	263	54.1
Pneumonia	Yes	186	38.3
	No	300	61.7
Septic Shock	Yes	10	2.1
	No	476	97.9
Anemia (pale conjunctives)	Yes	55	11.3
	No	431	88.7
Fever (altered body temperature)	Yes	227	46.7
	No	259	53.3
TB infection	Yes	18	3.7
	No	468	96.3
Malaria	Yes	54	11.1
	No	432	88.9
Measles	Yes	16	3.3
	No	470	96.7
HIV infection	Yes	8	1.6
	No	478	98.4
Edematous	Yes	151	31.1
	No	335	68.9

### Routine medication and supplementation

The most frequently prescribed regular medicines were intravenous antibiotics (85%) and amoxicillin (47.9%). Folic acid supplementation was given to 70.8% of children. About 34.8% of children under the age of two were eligible for deworming. Among the study participants, 32.9% and 10.1% had IV fluid and blood transfusions, respectively. Four hundred and sixty-one (94.9%), 88.8% and 80% of the admitted children were given F100 milk, F75 milk, and plumpy nuts, respectively (Table 4).

**Table 4:** Routine medication and supplementation of SAM children admitted to TFC of DPH, BGH, and MKCSH, from September 09, 2017, to April 04, 2021

Routine medication	Category	Frequency	percent
Amoxicillin (Oral)	Yes	233	47.9
	No	253	52.1
Folic acid	Yes	344	70.8
	No	142	29.2
Deworming	Yes	169	34.8
	No	317	65.2
Vitamin A	Yes	249	51.2
	No	237	48.8
Antimalarial drugs	Yes	42	8.6
	No	444	91.4
IV fluid	Yes	160	32.9
	No	326	67.1
IV Antibiotics	Yes	413	85.0
	No	73	15.0
Blood transfusion	Yes	49	10.1
	No	437	89.9
ResoMal	Yes	215	44.2
	No	271	55.8
Plumpy nut	Yes	389	80.0
	No	97	20.0
F100 Milk	Yes	461	94.9
	No	25	5.1
F 75 Milk	Yes	431	88.7
	No	53	10.9

### Treatment outcome and recovery rate

**Fig. 2:**
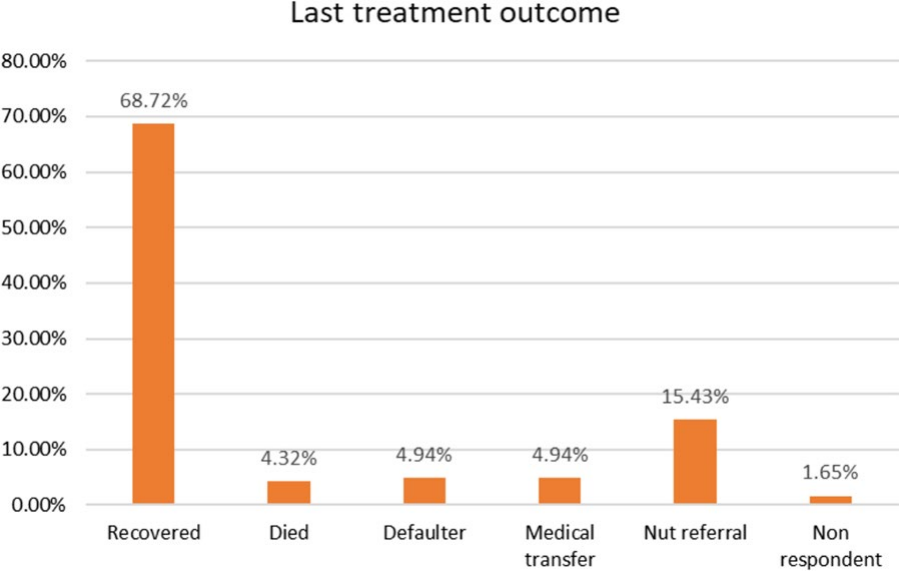
Treatment outcome of SAM children admitted to TFC of DPH, BGH, and MKCSH, from September 09, 2017, to April 04, 2021

Out of the 486 children, 68.72% were recovered, 4.32% died, 4.94% were both defaulters and medical transfers, 15.43% were transported out to the OTP center, and 8 (1.65%) were non-responders (Fig. 2). The median survival time was 21 days (95% CI 20.179, 21.821).

### Incidence of recovery from SAM and survival probability

**Fig. 3:**
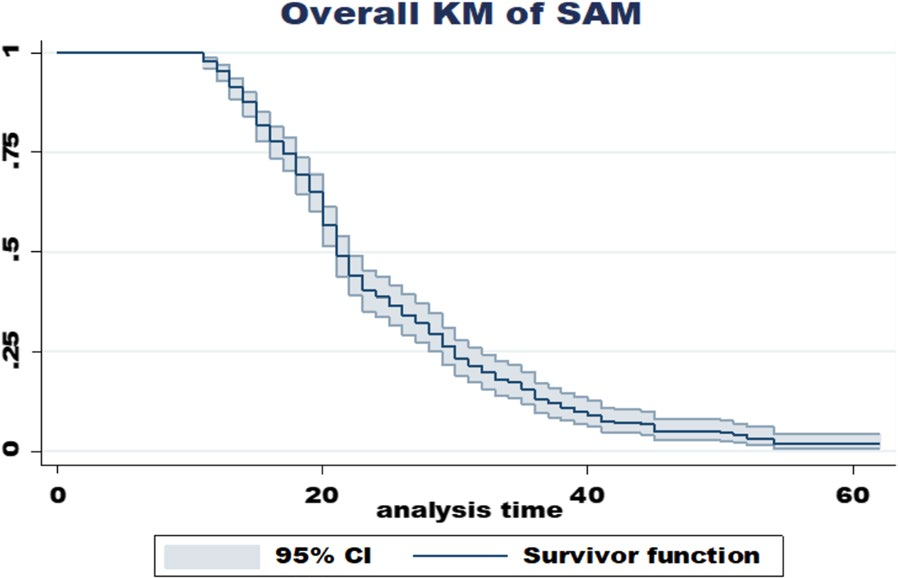
Overall Kaplan–Meier of survival curves for time to recovery from SAM among age 6–59 months children admitted to TFC of DPH, BGH, and MKCSH, from September 09, 2017, to April 04, 2021

At the end of the follow-up time, 68.72% (95% CI 64.8, 73) observations were recovered from SAM. The overall recovery rate was 3.35/100-person day (PD) with 95% CI of [3.010, 3.729] per 100 PD after total 9970 PD observation. At the institution, the recovery rates at DPH, BGH, and MKCSH were 2.68 (95% CI 2.30, 3.11), 5.12 (95% CI 4.27, 6.14), and 3.55 (95% CI 2.65, 4.75) per 100 PD, respectively. The cumulative survival probability of patients was 0.875 at 14 days, 0.491 at 21 days, 0.297 at 28 days, and 0.0212 at the end of the follow-up period (Fig. 3).

**Fig. 4:**
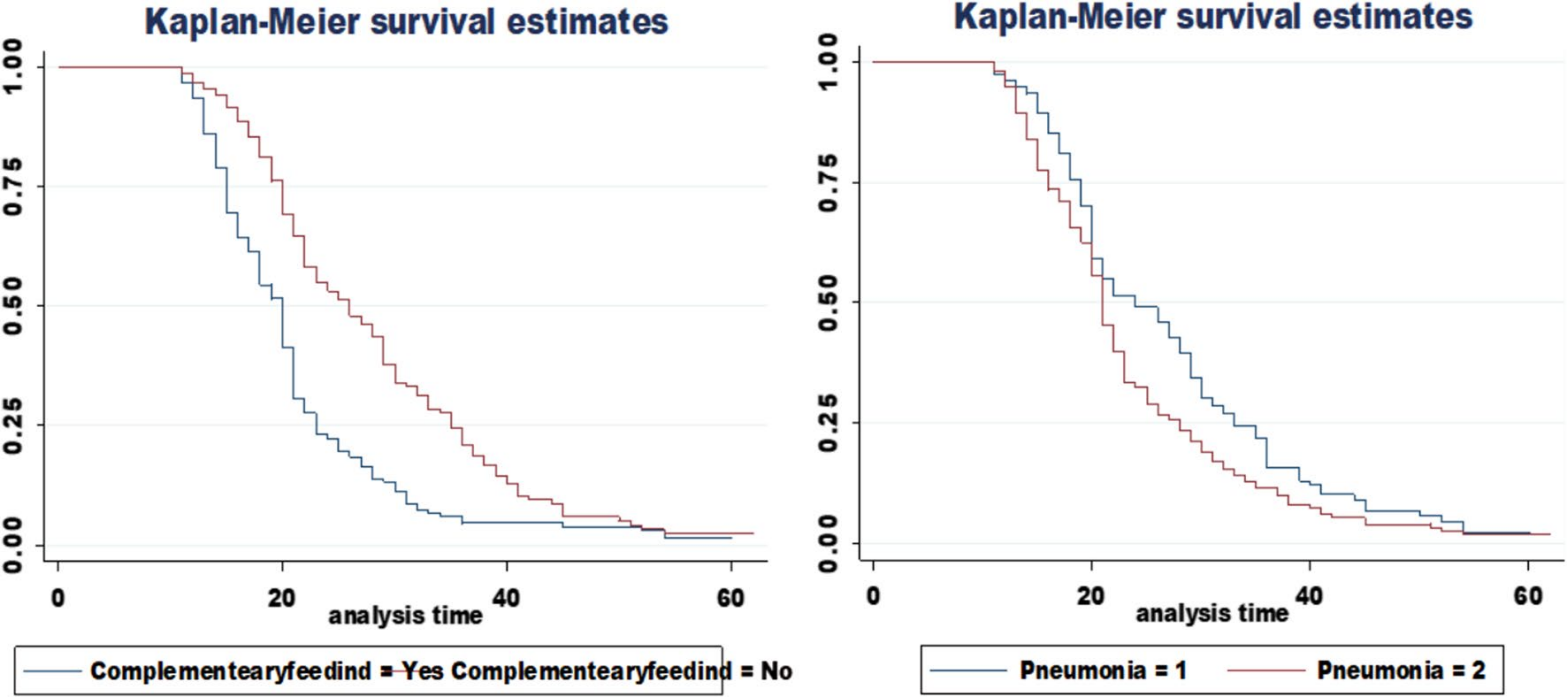
KM survival curve for complementary feeding and pneumonia for time to recovery from SAM among age 6–59 months children admitted to TFC of DPH, BGH, and MKCSH, from September 09, 2017, to April 04, 2021

To examine whether there is a difference in survival experience between members of the stated categories, a separate graph of estimations of the KM survival curve for each categorical variable has been created. The survivorship function pattern of those children who did not started complementary at six months and those with pneumonia has higher than their counterparts. It indicates that the group represented by the upper curve has a higher chance of surviving than the group represented by the lower curve which implies lower recovery rate of the upper curve group in Fig. 4.

### Variable selection and model diagnostics

**Fig. 5:**
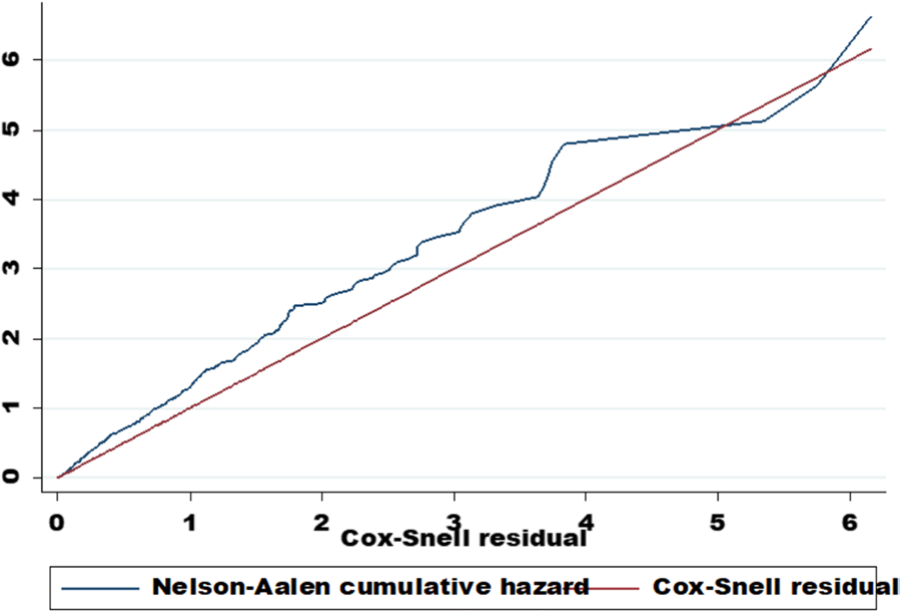
Final model adequacy graph based on Nelson Alan and Cox–Snell residual test, 2021

As variable selection precedes model diagnostics, during bivariate analysis, 12 variables that had a *P* value < 0.25 were selected for multivariable survival regression. The VIF ranged from 1.09 to 3.43 (Additional file 1: Table S1), which indicates the absence of multicollinearity. We checked PHA using the Schoenfeld residual test method (Global test, *X*^2^ = 38.52 with a p value of 0.080), which indicates the satisfaction of PHA (Additional file 1: Table S2). Using AIC, the Weibull parametric proportional hazard model was used as the best fit model (Additional file 1: Table S3). The cumulative hazard plot, which is derived from the Cox–Snell residual plot, follows a straight line through the origin with a slope of one, showing that the model’s goodness of fit is satisfactory (Fig. 5).

### Predictors of time to recovery from SAM

After adjusting for different variables, complementary feeding status of the child, pneumonia, amoxicillin, and folic acid supplementation were found to be independent predictors of recovery time of SAM children.

Children who had complementary feeding at six months were 1.44 times more likely to recover as compared to their counter parts (AHR = 1.44; 95%, CI 1.073, 1.935). Children who were admitted without pneumonia were 1.33 times more likely to recover as compared to those who had pneumonia at baseline as a comorbidity (AHR = 1.33, 95%, CI 1.049, 1.696). Children who took amoxicillin had a 1.31 (AHR = 1.31, 95%, CI 1.021, 1.685) times greater likelihood of fast recovery than their counterparts. Children with SAM who received folic acid were 1.82 times more likely to recover early (AHR = 1.82, 95% CI 1,237, 2.665) than children who did not receive folic acid (Table 5).

**Table 5:** Predictors of time to recovery from SAM in children admitted to Mettu Karl Specialized Hospital, Bedele General Hospital, and Darimu Primary Hospital Therapeutic Feeding Center Between September 09, 2017, to April 04, 2021

Variable	Category	Recovery		CHR (95% CI)	AHR (95% CI)
		Cured	Censored		
Residence	Rural	282	128	1	1
	Urban	52	24	1.46 (0.865, 1.011)	0.89 (0.635, 1.243)
Exclusive breast feeding	No	141	94	1	1
	Yes	193	58	1.33 (1.071, 1.654)	0.93 (0.708, 1.213)
Complementary feeding status	No	167	108	1	1
	Yes	167	44	1.98 (1.596, 2.456)	1.44 (1.073, 1.935) *
Fully Immunized	No	149	77	1	1
	Yes	185	75	0.85 (0.683, 1.052)	1.08 (0.836, 1.386)
Edematous at admission	Yes	97	54	1	1
	No	237	98	1.35 (1.063, 1.705)	1.23 (0.726, 2.073)
Pneumonia	Yes	125	61	1	1
	No	209	91	1.42 (1.136, 1.774)	1.33 (1.049, 1.696) *
Anemia	Yes	32	23	1	1
	No	302	129	1.46 (1.014, 2.102)	1.51 (0.863, 2.652)
Amoxicillin	No	13	80	1	1
	Yes	161	72	1.64 (1.322, 1.039)	1.31 (1.021, 1.685) **
Folic acid supplementation	No	114	28	1	1
	Yes	220	124	0.52 (0.418, 0.659)	1.82 (1, 237, 2.665) **
Vitamin A supplementation	Yes	160	77	1	1
	No	174	75	1.29 (1.037, 1.594)	1.17 (0.927, 1.479)
Blood transfusion	No	308	129	0.64 (0.429, 0.955)	1.13 (0.616, 2.056)
	Yes	26	23	0.64 (0.429, 0.955)	1.13 (0.616, 2.056)
Type of SAM	Marasmus	239	98	1	1
	Kwashiorkor	72	40	0.67 (0.512, 0.867)	0.87 (0.507, 1.509)
	Marasmic Kwashiorkor	23	14	1.27 (0.829, 1.958)	1.3 (0.652, 2.522)

**Significant association *P* < 0.01, *p value < 0.05

## Discussion

Despite the fact that there have been considerable improvements in child survival, SAM is still a concern in the healthcare system of developing countries like Ethiopia [34]. The present study revealed important information predictors of time recovery using a record of 486 children of age 6 to 59 months who were managed according to the SAM national treatment guideline [24].

The findings of the current study showed that the overall recovery rate from SAM is 68.72% (95% CI 64.8, 73) with incidence density of 3.35 per 100 PD with 95% CI of [3.010, 3.729] after total 9970 PD observation. This finding is below the minimum accepted international standard of 75% [33]. This finding is consistent with studies conducted in southern 69.2% [20] and Northwest 69.3% [22] Ethiopia. This finding is lower than the results of research carried out in various areas of Ethiopia, which range from 74.4% to 82.4% [19, 21, 28, 33, 35–37]. The increased patient flow to the study institutions from referrals from various districts and nearby regions, the high prevalence of comorbidities, and variations in how the SAM management recommendations were implemented could all contribute to these discrepancies. The variations in recovery periods may be partially explained by the late diagnosis of SAM and referral to stabilization centers [38].

The results of similar investigations carried out in Pawi General Hospital [8] and Aksum [39], where their findings were 65.8% and 56%, respectively, are greater than the current figure. This discrepancy can be attributable to the high caliber experts present in this study setup, including physicians and pediatric residents [21].

The current study revealed that children who had appropriate complementary feeding at age were more likely to recover from SAM compared with children who did not have it. This finding is supported by WHO guideline [40] and other studies [16, 41]. This could be because complementary foods are typically necessary to boost nutrient intake and supplement the typical diet [42]. The recovery of children with severe acute malnutrition has been aided by the use of supplemental diets with varied nutrient compositions, but their efficacy and effectiveness have fallen short of expectations [43].

This study also demonstrated that children with SAM who did not get pneumonia had a nutritional recovery rate that was noticeably higher than that of kids who did. Consistent with this finding, a hospital-based retrospective follow-up studies in Jimma [32], Addis Ababa [28], and WagHimra Zone, Northeast Ethiopia [44] found less recovery rate of SAM in comorbid of pneumonia children. The interaction between pneumonia and malnutrition, which has a synergistic effect, can be used to explain this [45].

According to this study’s findings on antibiotic therapy, children who received amoxicillin had a better likelihood of recovering from SAM than their counterparts. It was in line with a study carried out at the Jimma University Medical Center in southwest Ethiopia [21]. Similar findings from a randomized controlled trial on SAM children in Malawi demonstrated that nutritional recovery was faster for those who took amoxicillin than with those who received a placebo, as well as that the nutritional recovery period was shorter [46]. This may be due to the fact that amoxicillin reduces the frequency of common side effects of SAM, such as pneumonia and diarrhea [47]. Children who received folic acid supplements at the time of admission recovered faster than those who did not, according to the study. This result is congruent with one from an Ethiopian investigation of a comparable nature [48]. This may be because folic acid administration helps to prevent anemia, which further speeds up SAM recovery [49].

### Limitation of the study

Main strengths of the paper include the use of follow-up study design and advanced statistical analysis (Weibull regression analysis). This study had some limitations, such as the fact that all of its data came from secondary sources and there was no way to control the accuracy of the measurements made while the patients were in the hospital. Additionally, it was unable to look at the socioeconomic status and influences of the parents and guardians. Furthermore, this study did not take into account patient management-related variables including medical equipment and professional expertise that could have an impact on the outcome variable.

## Conclusions

The overall recovery rate from SAM fell short of the internationally recognized minimal standard (> 75%). Factors like complementary feeding status of the child, presence of pneumonia, treated by amoxicillin, and folic acid supplementation, significantly predict the recovery time of SAM. Therefore, it is recommended to concentrate on enhancing early detection and referral of SAM children to treatment facilities. While doing so, the provision of folic acid and amoxicillin, as well as the earliest possible treatment of concomitant conditions like pneumonia, should be emphasized by healthcare professionals in order to shorten SAM’s recovery time.

## Supplementary Information

**Additional file 1**. Model diagnostics for predictors of time to recovery from SAM among age 6-59 months children admitted at TFC of Southwest Ethiopia.

## Data Availability

Data will be available from the corresponding author upon request.

## Abbreviations

AHR: Adjusted hazard ratios
AIC: Akaike information criteria
BGH: Bedele General Hospital
DPH: Darimu Primary Hospital
HRs: Hazard ratios
HIV: Human immune deficiency virus
IV: Intravenous
KM: Kaplan–Meier
MKCSH: Mettu Karl Specialized Hospital
MUAC: Measurement of upper arm circumference
NG: Naso-gastric
PD: Person day
PHA: Proportional hazard assumption
ResoMal: Rehydration solution for malnutrition
SAM: Severe acute malnutrition
SSA: Sub-Saharan Africa
SD: Standard deviation
TB: Tuberculosis
TFCs: Therapeutic feeding centers
WFH: Weight for height
WHO: World Health Organization
VIF: Variance inflation factor
