# Supplementary material for: Severe acute malnutrition’s recovery rate still below the minimum standard: predictors of time to recovery among 6- to 59-month-old children in the healthcare setting of Southwest Ethiopia
Source: J Health Popul Nutr. 2022 Nov 4;41:48. doi: 10.1186/s41043-022-00331-9 (PMC9635096; doi:10.1186/s41043-022-00331-9)
Supplement: Supplementary file 1 — Additional file 1. Model diagnostics for predictors of time to recovery from SAM among age 6-59 months children admitted at TFC of Southwest Ethiopia. [file 41043_2022_331_MOESM1_ESM.docx]

Supportive results

**Table S1: Test of multicollinearity based on VIF for the covariates**

| **Variable** | **VIF** | **1/VIF** |
| --- | --- | --- |
| Edema | 3.43 | 0.291843 |
| SAM type | 3.42 | 0.292325 |
| Blood-transfusion | 1.66 | 0.603045 |
| Anemia | 1.61 | 0.620348 |
| Folic Acid | 1.48 | 0.676497 |
| Complementary feeding | 1.47 | 0.678308 |
| Breast feeding | 1.23 | 0.815178 |
| Immunization status | 1.21 | 0.823519 |
| Vitamin A | 1.14 | 0.878148 |
| Amoxicillin | 1.12 | 0.894547 |
| Residence | 1.11 | 0.898692 |
| Pneumonia | 1.09 | 0.921548 |
| **Mean VIF** | **1.62** | |

**Table-S2: Test of proportional hazard assumption based on Schoenfeld residuals for the covariates**

| Time: Time | Chi2 | df | Prob>Chi2 |
| --- | --- | --- | --- |
| Global | 38.52 | 15 | 0.080 |

**Table S3:** Model diagnostics based on AIC and BIC among SAM children admitted in therapeutic centers, southwest Ethiopia, 2021 (N=486)

| **Model** | **Df** | **AIC** | **BIC** |
| --- | --- | --- | --- |
| Cox | 14 | 3324.225 | 3382.832 |
| Exponential | 16 | 887.4388 | 954.4181 |
| **Weibull** | **17** | **454.546** | **525.7116** |
| Gompertz | 17 | 619.2771 | 690.4427 |

*Df=Degree of freedom, AIC = Akaike's information criterion, BIC= Bayesian information criterion*
